# Active Health Governance—A Conceptual Framework Based on a Narrative Literature Review

**DOI:** 10.3390/ijerph19042289

**Published:** 2022-02-17

**Authors:** Kuili Zhang, Bing Ran

**Affiliations:** 1 School of Public Administration, Central China Normal University, Wuhan 430079, China; zhkuili@ccnu.edu.cn; 2 School of Public Affairs, Pennsylvania State University, Middletown, PA 17057, USA

**Keywords:** active health governance, health policy, determinants of health, health system, governance for health, lifespan health, life-course health

## Abstract

Health policies are regarded as a governance mechanism crucial for reducing health inequity and improving overall health outcomes. Policies that address chronic conditions or health inequity suggest a governance shift toward active health over past decades. However, the current literature in health policy largely focused on some specific health policy changes and their tangible outcomes, or on specific inequality of health policies in gender, age, racial, or economic status, short of comprehensively responding to and addressing the shift. This is exacerbated further by a common confusion that equates health policy with health care policy, which has been burdened by increased population ageing, growing inequalities, rising expenditures, and growing social expectations. This study conducted a narrative literature review to comprehensively and critically analyze the most current knowledge on health policy in order to help us establish a theoretical framework on active health governance. The comprehensive framework proposed in this paper identifies the main elements of a well-defined active health governance and the interactions between these elements. The proposed framework is composed of four elements (governance for health, social determinants of health, lifestyle determinants of health, and health system) and three approaches (whole-of-government approach, whole-of-society approach, and lifespan/life-course approach) that are dynamically interacted to achieve two active health outcomes (health equity and health improvement). The framework provides a conceptual solution to the issues of current literature on health policy and practically serves as a new guide for health policymaking.

## 1. Introduction

Health policies aim at improving overall health outcomes and reducing health inequalities for the entire population through individual or collective health related intervention. To tackle challenges of our time, such as increased population ageing, heavy burden of chronic diseases, growing inequalities in health, uprising pressure from health expenditures, social expectations for better health conditions [1,2], health policies are experiencing a shift from disease-centered to active-health oriented. 

We can discern this shift from both health policy research and health policy making that pay greater attention to those challenges in general and chronic conditions and health inequalities in particular. First, strategies that policymakers can use to tackle chronic diseases are being developed. For example, self-management support focuses on the active participation of patients in their treatment through patient education [3], and integrated care focuses on improving linkage or coordination of services of different providers along the continuum of care [4]. Second, policies reducing health inequalities have undergone changes from centering on individual health behaviors to emphasizing the gradient and social determinants of health [5]. Some scholars view this policy change as a shift from “downstream” health determinants (medical care, environmental factors, and health behaviors) to “upstream” health determinants (education, income, social status, and general public policy) as the root causes of health disparities and illnesses [6]. Moving beyond the traditional health policy orientation, the shift to active health orientation emphasizes redesign and upstream intervention within and beyond the health system for purposes of reducing health inequalities, promoting wellbeing in the context of chronic conditions and containing the rising cost of maintaining healthy population. 

However, this shift from disease-centered to active-health-oriented is still far from being fully addressed in scientific debate. The current literature in health policy largely focused on specific health policy changes and their tangible outcomes, such as children, ageing, migrants, and mental health policies [7,8,9,10] or on specific inequality of health policies in gender, age, racial, or social-economic status [11,12], short of comprehensively responding to and addressing the shift. This is exacerbated further by a common confusion that equates health policy with health care policy. As a result, many countries have used “health policy” to denote “medical care policy,” which actually is only one variable in a nation’s health equation [13]. Moreover, most literature emphasizes a certain single health determinant at a time when studying structural determinants of health (economic, environmental, social, and cultural) or lifestyle determinants of health, while missing a more comprehensive framework to conceptualize the relationship between determinants. 

Taking these issues into consideration, this paper aims at developing a comprehensive conceptual framework of active health governance (health policy and administration) based on a narrative literature review. The research questions tackled in this article are as follows: *what are* *the main components of a well-defined active health governance and what are the interactions between these* *components*? Answering these two research questions is meaningful and contributes to the literature in three aspects: (1) the proposed comprehensive framework of Active Health Governance is distinctive from most of literature that had equated health policy to health care policy or dealt only with a subset of health determinants. (2) The literature on health policies and governance is dominated by micro-, medical-, hospital-, and program-orientated studies while our research puts forward a macro and systemic perspective towards health policies and governance. (3) Active Health Governance could be viewed as a new approach or strategic solution that could produce more health equality and better health outcomes if it is put into practice. We also realized the limitation of this research, which includes at least three aspects: (1) It is not based on practices performed by countries but based on the conceptualization of the literature reviewed that encompassed “active” elements within health policies. (2) It is difficult to measure the performance of every element within the framework due to the limited operationalizations of these elements, which further results in difficulties in evaluating the elements’ contributions to health outcomes. (3) It is not easy to implement this framework due to comprehensive nature of this framework—indeed, active health governance requires all stakeholders, such as governments, private companies, non-governmental organizations, civil society, and individuals, to take shared responsibilities toward active health.

In the next sections, we will discuss the narrative literature review methodology used in this paper and propose a framework ingrained in the literature. The framework we developed on “active health governance” is driven by whole-of-government and whole-of-society approaches and pays close attention to upstream causes of health (e.g., social determinants of health) while promoting individual lifestyle and improving health care delivery. We will then delineate three interactive elements in this framework of active health governance—social determinants of health, lifestyle determinants of health, and health system targeting at people’s every critical life stage—toward the goals of health equity and overall health improvements. The article concludes with a discussion on the contributions of the proposed framework and future research directions built upon the framework. 

## 2. Methods

In this study, we conducted a narrative literature review, a literature review methodology informed by Greenhalth [14] Lloyd [15], Bower and Gilbody [16], Watters et al. [17], Shachak and Reis [18], and Humphries et al. [19]. The goal of our review is to comprehensively and critically analyze the most current knowledge on health policy in order to help us establish a theoretical framework on active health governance. 

For our narrative literature review, we considered carefully which database to use to retrieve literature on heath policy. We considered Google Scholar, MEDLINE, PubMed, and CINAHL but found these databases to be either too general to set up the search criteria (Google Scholar) or too specific on biomedical literature (MEDLINE and PubMed) or nursing literature (CINAHL), rather than on health policy literature. We finally decided to make use of Web of Science that includes SCI, SSCI, A&HCI, CPCI-S, CPCI-SSH, BKCI-S, BKCI-SSH, ESCI, CCR-EXPANDED, IC. Indexes. Using the search term “health policy” and “health system” (with the quotation marks) to search titles, abstracts, and keywords of all articles indexed in Web of Science identified 1908 potentially relevant studies. The following inclusion/exclusion criteria were applied: (1) peer-reviewed journal articles while excluding book chapters, conference proceedings, etc. We believe peer-reviewed articles have been scrutinized by editors and reviewers in the field and, thus, provide balanced perspectives. (2) Those published in English between 2010 and 2020 since we are primarily interested in health policy changes in recent decades and a search spanning a period of the most recent decade provides us with a well-rounded understanding of the newest development in the literature and enables the identification of key themes persisting over this decade of research. (3) We focused on health policy services or health care sciences or primary health care, rather than psychiatry, biomedical, nursing, medical informatics, obstetrics gynecology, etc. In summary, the search query is as follows: TOPIC: (“health policy” AND “health system”); refined by: WEB OF SCIENCE CATEGORIES: (“health policy services” OR “health care sciences services” OR “primary health care”) AND DOCUMENT TYPES: (article); timespan: 2010–2020; indexes: SCI-EXPANDED, SSCI, A&HCI, CPCI-S, CPCI-SSH, BKCI-S, BKCI-SSH, ESCI, CCR-EXPANDED, IC.). The inclusion/exclusion process identified 413 articles. 

After retrieving the selected 413 articles, two authors independently performed an evaluation of the abstracts of these articles on whether the articles are targeted at health policy systems. Any discrepancies between the two researchers were resolved by using discussions. After this step, we deleted articles that focused on medical or pharmaceutical issues or diseases (e.g., mental/psychological health), which further narrowed our dataset down to 124 articles. Our final review included these 124 articles. 

We then coded the 124 articles. The coding has three steps. In step 1, each author read the full text of each article to extract the main themes and topics discussed in each article. We periodically exchanged notes on our summary of major themes of each article and discussed any differences until an agreement was reached. In step 2, we analyzed the coded themes and topics and synthesized them into higher-level categories by identifying initial relationships among them. This is the transition from the initial codes in step 1 to theoretical categories. For example, “patient-centered care,” “patient activation,” “integrated care,” and “community-based care” are all synthesized into the category of “Health System.” In step 3, we compared and contrasted the theoretical themes in step 2 to understand the roles they play vis-a-vis one another and to look for conceptual linkages between these theoretical themes so that we can abductively construct a framework. This resulted in our final construction of a comprehensive framework on Active Health Governance. 

Next, we will report the major themes in health policy research that we summarized. Then, we present the framework we constructed that systemizes major themes in the literature with four elements and three approaches that are dynamically interacted to achieve two active health outcomes. The framework provides a conceptual solution to the issues of current literature on health policy and practically serves as a new guide for health policymaking and health administration. 

## 3. Major Themes in Literature

Five major themes emerged from the reviewed articles on health policy system. We will first discuss each of these major themes as we summarized from the literature in this section. Then, in the next section, we propose our framework on active health governance derived from these themes. In our discussion of major themes, a series of abbreviations are used: Patient-Centered Care (PCC); Patient Activation (PA); Integrated Care (IC); Community-based Care (CBC); Social Determinants of Health (SDH); Lifestyle Determinants of Health (LDH); and World Health Organization (WHO).

### 3.1. Patient-Centered Care (PCC) and Patient Activation (PA)

PCC focuses on taking individuals, families, and communities’ perspectives and is determined by the quality of interactions between patients and clinicians through which health system performance could be improved [20]. Thus, PCC emphasizes the role that patients play in high-quality patient care, and the patient role is now treated as an important dimension in care systems [20,21,22]. Growing evidence shows that PCC improves disease outcomes and quality of life and provides more efficient care and improved population health [20,23]. The literature generally agree that PCC embodies “a shift from a traditional, paternalistic, provider-driven and disease-focused approach towards one that fully integrates the patient’s perception, needs and experiences, into every phase of medical consultation, treatment and follow-up” [24].

PA is defined as “understanding one’s own role in the care process and having the knowledge, skills, and confidence to take on that role” [25]. PA involves four stages: “(1) believing the patient role is important, (2) having the confidence and knowledge necessary to take action, (3) actually taking action to maintain and improve one’s health, (4) staying the course even under stress” [26]. PA sometimes is treated as a subset of the patient empowerment, where the former focuses on specific diseases or programs and the latter focuses on a general emergent state [27]. Increased evidence showed that PA is associated with higher self-management behaviors [25,26,28], higher quality-of-life scores, and lower patient care costs [29]. 

### 3.2. Integrated Care (IC) and Community-based Care (CBC)

According to World Health Organization (WHO), IC should be centered on the needs of individuals, their families, and communities [30,31]. IC was proposed to address care fragmentation in healthcare delivery because IC can “bridge the boundaries between professions, providers and institutions thus better support the rising number of people with chronic health problems” [32]. Some countries have developed national IC strategies covering the entire care continuum from health promotion to care for complex chronic disease patients [33,34,35]. Key elements of IC include “policies on improved collection and sharing of information, moving care into the community and aligning payment schemes to incentivize care coordination and enhance integration of provision of services” [35]. However, the overall benefit of IC is rather mixed as reflected in the literature [36,37].

Community-based care (CBC) is the rebalance of health systems from institution-based to community-based. Since 1990s, “many European countries abandoned the traditional orientation towards a health system almost exclusively oriented to treat illness through high-tech hospital-based services and rekindled the approach to some form of CBC system” [34]. Scholars believe that CBC could “improve care in the community, through strengthened primary care as well as strengthened community-based care” [38]. It is characterized by “the use of epidemiology and clinical skills to depict the health needs of the community, assuming responsibility for a defined population, clear-defined programs to address communities’ health needs, community involvement, and accessibility to services” [38,39]. 

### 3.3. Social Determinants of Health (SDH)

In order to tackle health inequalities, SDH such as income, wealth, and education, etc., have been highlighted and viewed in the literature as causes of inequalities [40,41]. Growing scientific evidence from promising interventions focused on SDH, such as “education and early childhood intervention, urban planning and community development, housing, income enhancements and supplements, and employment” [42,43,44,45] has been proved to be able to improve population health and reduce health disparities [44,45,46,47]. Scholars and policy makers have proposed that some social factors such as “taxation and tax credits, old-age pensions, sickness or rehabilitation benefits, maternity or child benefits, unemployment benefits, housing policies, labor markets, communities, and care facilities” [48,49] are necessary social determinants of health.

Of particular importance is the alarming impact of racism and racial discrimination in health policy. Racism stems from the belief that people should be treated differently because of a few phenotypic features. Racism can manifest as individual or group acts and attitudes or institutionalized processes that result in health disparities (differential access to health services; poorer psychological and physiological wellbeing of the minorities; racial prejudice that undermines the doctor-patient relationship, etc.) [50,51]. Researchers have identified different forms of racism within health policy and how racism is associated with morbidity and mortality [52]. Many scholars have argued that racism is a structural and long-standing system that can be eliminated only with a sustained, multilevel, and interdisciplinary approach [53].

Marmot proposed the theory of social gradient that not only suggests that health inequalities are a problem that affects entire populations [54] but also implies that actions on the social determinants of health must involve whole-of-government and whole-of-society approaches. Therefore, coherent actions across government, at all levels, are essential for improving health equity: “Intersectoral action for health—coordinated policy and actions among health and non-health sectors—can be a key strategy to achieve policy coherence [55]. Meanwhile, reaching beyond government to involve civil society and the voluntary and private sectors is vital for health equity and can help to ensure fair policy making” [56]. 

### 3.4. Lifestyle Determinants of Health (LDH)

LDH focus on changes in individual behavior and lifestyle. It includes “safe sexual behavior and reproductive health, physical activity, eating habits and safe food, as well as reductions in tobacco and alcohol consumption, drug use, and excessive gambling” [13]. Research findings suggest that lifestyle behaviors are important in addressing diseases such as obesity, diabetes, cardiovascular disease, and even mortality [57,58,59]. Accordingly, extensive literature focused on the evaluation of public health policies that may produce and keep healthy lifestyle or change unhealthy lifestyle. Public interventions mainly involve the following policies on lifestyle determinants of health: policies on tobacco control [60,61,62,63], policies on alcohol, sugar-sweetened beverage, food and nutrition [64,65,66,67,68], and promoting physical activity [69,70,71,72].

### 3.5. Lifespan/Life-Course Health

Literature recognized that early life conditions affect the emergence and evolution of human traits, which affect a variety of outcomes in adult life, including health [73,74,75,76]. The research on lifespan/life-course perspective focuses on understanding how early-life experience can shape health across the entire lifetime, particularly adult chronic diseases and their risk factors and consequences, and generations [74]. The research of lifespan/life-course approach extends it to all age groups, covers health topics in different countries, and advances into health strategies and programs. Kuruvilla et al. provided a conceptual framework for a lifespan/life-course approach to health composed of four stages: “(1) birth, the neonatal period and infancy; (2) early and later childhood and adolescence; (3) youth and adulthood; and (4) older adulthood” [73]. In this framework, “functional ability and intrinsic capacity are depicted as idealized arcs across the lifespan/life course. Whereas intrinsic capacity follows a biologically determined trajectory of physical and mental capacities, functional ability can be optimized throughout life by a supportive environment” [77]. Although there is substantial literature on the lifespan/life course approach, the translation of life course theory and research into practices is far from fully developed [75].

## 4. A Proposed Framework on Active Health Governance

The narrative review of literature not only generates significant themes that scholars have fruitfully researched on health policy but also points to some issues in the current conceptualization of health policy. We noted that articles we reviewed largely focusing on specific health policy changes and their tangible outcomes by concentrating on certain elements of a theme at a time or focusing on general inequalities of health policies, short of comprehensively responding to and addressing the shifting focus in health policy and the dynamic interactions between major themes. We also observed that most literature emphasizes a certain single health determinant at a time while missing a more comprehensive framework to conceptualize the relationship between structural determinants of health or lifestyle determinants of health. There is clearly a dire need for a comprehensive framework on active health governance to integrate our current understanding on active health and to conceptualize the dynamic interactions among major factors for active health. 

**Figure 1:**
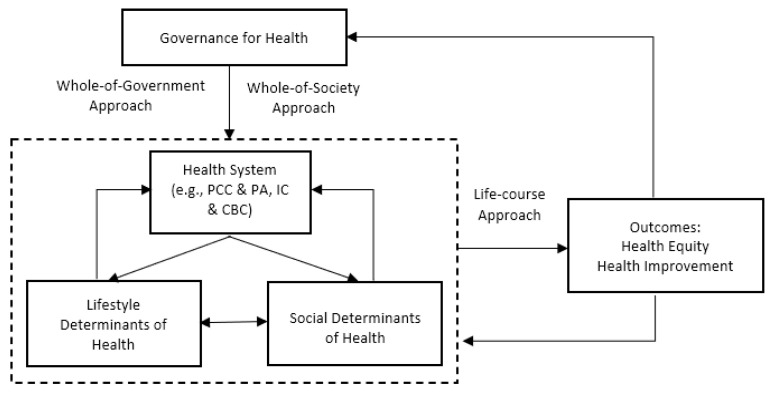
A proposed conceptual framework on active health governance.

Built upon our findings of major themes in the literature, we constructed a framework on active health governance. We define *active health governance as health policy and administration that adopts whole-of-government and whole-of-society approach to link all health-influencing factors and provides targeted interventions according to people’s critical life stages in order to achieve equitable and better health outcomes*. This conceptual framework on active health governance that we propose is composed of four elements, three approaches, and two outcomes (Figure 1).

### 4.1. Elements of Active Health Governance and Their Interplays

The first element of the framework is governance for health. Governance for health was defined as “the attempts of governments or other actors to steer communities, countries or groups of countries in the pursuit of health as integral to well-being through both whole-of-government and whole-of-society approaches” [78]. The concept of governance for health can best be illustrated as “the culmination of three waves in the expansion of health policy—from intersectoral action, to healthy public policy to the health in all policies (HiAP) approach—all of which are now integrated in whole-of-government and whole-of-society approaches to health and well-being” [78,79]. 

The whole-of-government approach is “an umbrella term describing a group of responses to the problem of increased fragmentation of the public sector and an imperative to increase integration, coordination and capacity” [80]. At the core of the whole-of-government approach is the joint cooperation across different governmental agencies and branches. It stresses the need for better coordination and integration, centered on the overall societal goal for which the government stands [79]. The whole-of-society approach is “a form of collaborative governance that emphasizes coordination through normative values and trust-building among a wide variety of actors” [78]. The whole-of-society approach is derived from the whole-of-government approach because wicked problems such as obesity and pandemic preparedness usually “require more than the whole-of-government approach: solutions require involving many social stakeholders, particularly citizens” [81]. Both whole-of-government and whole-of-society approaches are intertwined with each other and mutually complementary. Moreover, both approaches contribute to shared governance for health towards improving health for all and reducing health inequalities.

The core of the active health governance is composed of three elements: health system, social determinants of health, and lifestyle determinants of health [82,83,84,85]. The health system is defined by WHO as all the activities for which their primary purpose is to promote, restore, or maintain health. In recent years, researchers and policy makers have proposed PCC, PA, IC, CBC, etc. as the various innovative forms within health system. By carrying out four vital functions—service provision, resource generation, financing, and stewardship—the health system aims at achieving “three fundamental objectives—improving the health of the population they serve, responding to people’s expectations, and providing financial protection against the costs of ill-health” [86]. WHO strongly advocates to strengthen health systems to mitigate current conventional health systems: hospital-centrism, commercialization, and fragmentation [87,88].

Social determinants of Health (SDH) and lifestyle determinants of health (LDH) are the other two elements of the core. Among those factors affecting people’s health, SDH acts as the predominant role that influences individuals’ health behavior through material and psychosocial mechanisms. LDH, on the other hand, mainly concerns individual behaviors influenced by social determinants and/or health systems, especially shaped by public health policies. Clearly, lifestyle determinants are closely associated with and shaped by social determinants. It can be viewed that “adoption of health-threatening behaviors is a response to material deprivation and stress. Environments determine whether individuals take up tobacco, use alcohol, have poor diets and engage in physical inactivity. Tobacco and excessive alcohol use, and carbohydrate-dense diets, are means of coping with difficult life circumstances” [82]. Lifestyle elements, in turn, have an active connection with social determinants of health. Different individuals have varying choices of behaviors under the same circumstances. The counteraction of health-relevant behaviors to socioeconomic status has been demonstrated in some studies [83,84]. These studies suggest that previous research may have underestimated the influence of health behaviors to social inequalities and are important in contributing to health differences [83,85]. Therefore, lifestyle determinants have a counter effect to circumstances around individuals, ultimately play an important role in health maintenance. 

Health system dynamically interacts with social and lifestyle determinants of health. For example, health determinants affect health system performance through a series of public policies, among which, the most powerful one includes welfare redistributive policies (or the absence of such policies) [42]. Individuals who are trapped in poverty are less likely to access to health care, more likely to suffer from catastrophic health expenditure, and result in adverse exposure and vulnerability, compared to those who stay at top of the social gradient. Moreover, the health system can improve unbiased access to care and improve the health status of citizens. The health system also influences social/lifestyle factors by mediating the differential consequences of illness in people’s lives. The role that health care plays in contributing to health differences “is increasing due to better results in disease prevention, improved diagnostic tools and treatment methods” [89]. Thereby, health care might decrease or increase health differences between socioeconomic groups depending on whether it is distributed pro-poor or pro-rich in different service chain in relation to need [84].

Investing in health requires investing in the health system and social and lifestyle determinants by using the lifespan/life-course approach: “Social arrangements and institutions (preschool, school, the labor market and pension systems) have a huge effect on the opportunities that empower people to choose their own course in life” [41]. Strategies for intervention to health inequalities and social determinants can be adapted to cater for the most important stages of the lifespan/life-course, such as the following: (1) maternal and child health. It requires not only a promotion of excellent health care in prenatal, perinatal, and postnatal periods but also a broad range of social policies such as “employment and social protection system that recognizes the risks posed by poverty and stress in early childhood, good parental leave arrangements, support for parenting and high-quality early education and care” [43] and universal access to primary and secondary school provision. (2) The second stage includes healthy adults. Material deprivations from unemployment or low-paid work and feelings of unfair pay in organizations with high levels of wage disparity contribute to physical and mental ill health. Occupational position is important for people’s social status and social identity, and threats of job instability affect health and wellbeing [43,90]. (3) The third stage includes healthy older people. Healthy aging requires managing the development of chronic morbidity and improving survival and wellbeing through health systems. It also requires the development of age-friendly policies and supportive environments to enable senior citizens’ full participation in community and society through a variety of factors, including fiscal, social welfare, transport, urban planning, housing, justice, and education. 

The four elements and the three approaches in the framework work jointly to impact health outcomes—health equity and health improvement. Health outcomes, in turn, can feed back to the four elements and the three approaches. Inequity in health and ill health, for instance, can negatively impact individual’s social determinants “by compromising employment opportunities and reducing income. Certain epidemic diseases can affect the functioning of social, economic and political institutions” [42]. By contrast, health equity and better health outcomes can facilitate better governance for health and promote the conditions of SDH, LDH, and health system by acquiring occupational position, community participation and social cohesion.

### 4.2. Characteristics of the Proposed Framework on Active Health Governance 

The proposed conceptual framework on active health governance demonstrates at least five characteristics when it is embedded in the literature we reviewed.

First of all, it is a comprehensive and holistic framework encompassing all active factors distilled from the literature and systemizing the dynamic interactions between these factors. We noted that policies or programs that only focus on one active factor can hardly achieve desirable health outcomes. Addressing integrated health services alone, for instance, while disregarding social and lifestyle determinants of health, is difficult to achieve the goals of improving health outcomes. Highlighting interactions of those factors within the framework, rather than overemphasizing a certain single element, is a prominent characteristic of this active health governance framework.

Second, it adopts a collaborative governance perspective [91,92], e.g., whole-of-government and whole-of-society approaches. It recognizes that health equity and health improvement depend on collaboration between all levels of governments and all societal actors such as businesses, civil society, and citizens, in which all actors take shared responsibilities, form partnerships, and work together to achieve active health goals [93]. Health is not an independent sector—it is closely linked with other complex adaptive systems with many spillover effects [78]. Most importantly, this framework recognizes that the private sector, civil society, communities, and individuals are frequently required to engage in this active health process. Although the government is often the leader or broker, other actors, such as a strong non-governmental organization or an alliance or coalitions of organizations, can also lead and play critical roles in active health [81]. 

Third, citizen empowerment is essential for active health governance. Empowerment is recognized as the combination of ability, motivation, and power opportunities [27]. For example, patient empowerment includes not only patient activation and patient participation in decision making with their health providers but also changing people’s role from passive care recipients to active agents with power and control, covering the action scope from care services to all health-related policies. By acquiring ability and motivation, citizens can be mobilized and involved in co-producing active health.

Fourth, the proposed framework emphasizes the importance of the lifespan/life-course approach to achieve health outcomes. Interventions should be different for childhood, adulthood, and older adulthood for different lifespans. The gender and race/ethnicity are other factors of targeted intervention. The policy towards older women, for instance, should be distinctive from older men because they always live longer with chronic conditions and are more likely to suffer from deprivation and social discrimination. Thus, the lifespan/life-course approach provides relevant interventions to health inequalities and social determinants for key stages of the lifespan/life-course in order to achieve active health outcomes. 

Lastly, the framework highlights upstream intervention of health while seeking for treatment of ill-health. The shift in health policies from a “downstream” approach to “upstream” one reflects our awareness that active health governance should address both the care for the sick and injured and the protection of health for the entire population. The framework emphasizes research on the social determinants of health, promoting policies for protecting health rather than simply improving health care, and intervening at the level of health systems rather than health professionals [94]. From this point, this framework on active health governance ought to be viewed as a “proactive” strategy that pays more attention to the root causes for ill health.

## 5. Discussion 

The existing research on health policy is mainly focused on specific populations, specific diseases, specific health policy changes and their tangible outcomes, or on the unequal status of health policies in different ages, gender, racial, social status, etc. Most literature emphasizes a certain single health determinant at a time or overemphasizes the function of certain elements, short of considering the contributions of other factors. Moreover, previous studies on determinants of health are frequently centered on one-way influence produced by social or lifestyle determinants of health while the counter influences made by individual behaviors or health system were relatively ignored. This is exacerbated further by a common confusion that equates health policy with health care policy that narrowed the understanding or resulted in a misunderstanding of concept of health policy.

The framework of active health governance proposed in this paper is built upon our detailed narrative analysis of the major themes discussed in literature and places emphasis on citizens’ potential to be responsible for their own health, while being protected from health care services based on citizen’s health rights, which is not limited to health care episodes. It highlights targeted interventions of health that are not only based on each critical life span but also on gender, race/ethnicity, and other disparities among or within groups. Therefore, this framework goes beyond the scope of the lifespan/life-course approach, seeking to provide targeted interventions according to people’s unique characteristics, towards a more effective supply of health services. 

One critical aspect in active health governance is the use of artificial intelligence (AI), which poses both opportunities and risks to health-related issues. AI, on the one hand, has enormous potential for health and are already used widely in delivery of health care, health systems management and planning, public health surveillance, and in health research. On the other hand, AI could expand existing inequalities in health care and systems based on race, ethnicity, age, gender, and social/economic status to the degree to enlarge digital divide for health. In order to tackle these potential risks, ethical considerations and human rights, such as protecting autonomy, promoting human wellbeing and public interest, fostering responsibility and accountability, and ensuring inclusiveness and equity, must be placed at the center of the design, development, and deployment of AI technologies for health [95,96]. Guided by appropriate ethical norms and standards, all human beings can equally benefit from the promise of AI technologies in the future.

By providing details on the interactive relationship among key elements impacting health and adding a feedback loop between outcomes and process, this framework on active health governance also moves beyond comprehensive primary health care proposed by WHO. Furthermore, it is closely associated with active social policy that mainly includes labor market policies, children’s policies, and aging policies. Active health governance, however, is distinctive from the active social policy because it demands a more exhaustive shift of policies. Within this framework, a series of significant changes in stakeholders is required: for individuals, the shift from passive receiver of care services to co-producers of health; for health care providers, the shift from diseases treatment to prevention and health promotion; and for governments and society, the shift from fragmentation of sectors to collaborative governance for health. Active health governance, in brief, can be considered as a genuine shift from “downstream” to “upstream” intervention for health. Translating this approach into actions, however, will involve overcoming numerous barriers and further efforts, both theoretically and practically.

## 6. Conclusions

Built upon a detailed narrative analysis of the major themes discussed in health policy literature, we proposed a framework on active health governance that could provide a conceptual solution to the issues of current literature on health policy, and practically serve as a new guide for health policymaking. It could be considered as an approach or strategy that is expected to address the health policy challenges faced by any countries. Within this framework, health-influencing factors of social determinants of health, lifestyle determinants of health, and health system are mutually interacting with each other and targeted at individual’s each critical life stages, driven by collaborative governance by multi-stakeholders, for the purposes of reducing health inequity and improving health outcomes. This framework clarifies the understanding of health policy, advocates a macro, interdisciplinary and comprehensive research perspective, triggers a shift of health strategies, and facilitates tackling those old challenges that most countries still face. We envisage that future research could focus on case studies of such holistic and comprehensive health policies reform; or to explore mechanism of interactions among elements of this framework (governance for health, health system, social determinants, and lifestyle determinants of health); and, correspondingly, to evaluate the performance of health governance using this framework.
